# Fn14 and TNFR2 as regulators of cytotoxic TNFR1 signaling

**DOI:** 10.3389/fcell.2023.1267837

**Published:** 2023-11-06

**Authors:** Daniela Siegmund, Olena Zaitseva, Harald Wajant

**Affiliations:** Division of Molecular Internal Medicine, Department of Internal Medicine II, University Hospital Würzburg, Würzburg, Germany

**Keywords:** apoptosis, Fn14, necroptosis, TNF, TNFR1, TNFR2, TRAF2, TWEAK

## Abstract

Tumor necrosis factor (TNF) receptor 1 (TNFR1), TNFR2 and fibroblast growth factor-inducible 14 (Fn14) belong to the TNF receptor superfamily (TNFRSF). From a structural point of view, TNFR1 is a prototypic death domain (DD)-containing receptor. In contrast to other prominent death receptors, such as CD95/Fas and the two TRAIL death receptors DR4 and DR5, however, liganded TNFR1 does not instruct the formation of a plasma membrane-associated death inducing signaling complex converting procaspase-8 into highly active mature heterotetrameric caspase-8 molecules. Instead, liganded TNFR1 recruits the DD-containing cytoplasmic signaling proteins TRADD and RIPK1 and empowers these proteins to trigger cell death signaling by cytosolic complexes after their release from the TNFR1 signaling complex. The activity and quality (apoptosis *versus* necroptosis) of TNF-induced cell death signaling is controlled by caspase-8, the caspase-8 regulatory FLIP proteins, TRAF2, RIPK1 and the RIPK1-ubiquitinating E3 ligases cIAP1 and cIAP2. TNFR2 and Fn14 efficiently recruit TRAF2 along with the TRAF2 binding partners cIAP1 and cIAP2 and can thereby limit the availability of these molecules for other TRAF2/cIAP1/2-utilizing proteins including TNFR1. Accordingly, at the cellular level engagement of TNFR2 or Fn14 inhibits TNFR1-induced RIPK1-mediated effects reaching from activation of the classical NFκB pathway to induction of apoptosis and necroptosis. In this review, we summarize the effects of TNFR2- and Fn14-mediated depletion of TRAF2 and the cIAP1/2 on TNFR1 signaling at the molecular level and discuss the consequences this has *in vivo*.

## 1 Introduction

**FIGURE 1:**
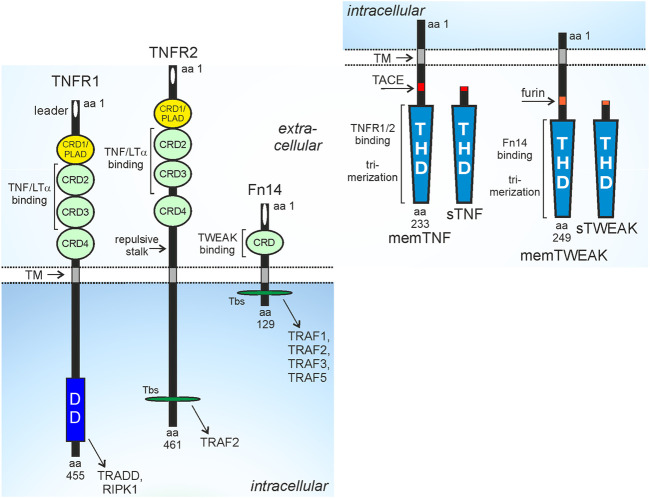
Domain architecture of TNFR1, TNFR2, Fn14 and their ligands. CRD, cysteine-rich domain; DD, death domain; PLAD, pre-ligand binding assembly domain; THD, TNF homology domain; TM, transmembrane domain; Tbs, TRAF binding site; protease processing sites for TACE and furin are indicated by red and orange rectangles. For more details see main text.

Tumor necrosis factor (TNF) receptor-1 (TNFR1), TNFR2 and fibroblast growth factor inducible 14 (Fn14) belong to the TNF receptor superfamily (TNFRSF) (Locksley et al., 2001). The receptors of the TNFRSF (TNFRs) can be categorized in three groups based on functional and/or structural differences. The TNFRs of the first group are defined by containing a structurally conserved protein-protein interaction domain in their cytoplasmic part called death domain (DD). The DD enables the DD-containing TNFRs, often also called death receptors (DRs), to recruit DD-containing cytoplasmic proteins by DD-DD interactions (Locksley et al., 2001; Siegmund et al., 2017). The DR-interacting cytoplasmic DD-containing proteins include enzymes, such as the kinase RIPK1, but also adapter proteins, such as TNFR1-associated death domain (TRADD) and Fas associated death domain (FADD) which enable the secondary recruitment of the E3 ligase TRAF2 or the initiator caspases caspase-8. The cytoplasmic DD-containing proteins link the DRs to signaling pathways eventually triggering cell death programs, in particular extrinsic apoptosis and necroptosis. While induction and execution of apoptosis is based on activation of proteases of the caspase family, induction and execution of necroptosis require kinase signaling and plasma membrane pore forming proteins (Bao and Shi, 2007; Shalini et al., 2015; Flores-Romero et al., 2020). Importantly, the extrinsic apoptosis signaling-inducing protease caspase-8 not only triggers the execution phase of apoptosis by direct processing of effector procaspases, e.g., procaspase-3 and cleavage of BID resulting in the additional stimulation of intrinsic caspase-9-dependent apoptosis pathway but also inhibits necroptotic signaling by cleavage of RIP kinase 1 (RIPK1). As a consequence apoptosis is the dominant default cell death mechanism in DR signaling (Schwarzer et al., 2020). Apoptosis typically does not result in inflammatory reactions *in vivo*, while necroptosis results in inflammation due to the release of damage-associated molecular patters (DAMPs) from dying cells and subsequent DAMP-mediated activation of immune cells (Bedoui et al., 2020; Newton et al., 2021). Thus, dependent on the cell death program engaged, DR-induced cell killing can have physiologically quite different consequences. Besides cell death, the DRs also activate the NFκB and MAPK signaling pathways under crucial involvement of the aforementioned DD proteins (Siegmund et al., 2017). The receptors of the second group of the TNFRSF also engage NFκB and MAPK signaling but have no DD and do not signal by help of DD-containing proteins. Instead, these TNFRs directly recruit TRAF2 and other proteins of the TRAF family by short TRAF-interacting motifs. The third group of TNFRs comprises soluble and glycosylphosphatidylinositol-anchored molecules as well as receptor variants with a nonfunctional DD and shares their ligands with receptors of the two other groups. The receptors of the third group of the TNFRSF accordingly act as decoy receptors. While TNFR1 is a DR, TNFR2 and Fn14 belong to the TRAF-interacting subgroup of the TNFRSF (Figure 1).

TNFR1 is ubiquitously and constitutively expressed while the expression of TNFR2 and Fn14 is more restricted and dynamic. TNFR2 is regularly found at high levels on myeloid cell types, regulatory T cells (Tregs), activated CD8^+^ T cells and B cells but is also expressed by resting T cells, type 2 innate lymphoid cells (ILC2), mesenchymal stem cells and endothelial and epithelial cell types (Hurrell et al., 2019; Medler and Wajant, 2019). Proinflammatory cytokines, furthermore, can further enhance TNFR2 expression (Winzen et al., 1993; Wang et al., 2006; Al-Lamki et al., 2008; Hurrell et al., 2019; Medler and Wajant, 2019; Suto et al., 2022). During development Fn14 is strongly and temporally expressed but later, in the fully developed organism, Fn14 expression is restricted to various types of pluripotent and unipotent stem cells (Wang et al., 2022). Worth mentioning is, however, that a plethora of growth factors, cytokines, pathogen-associated molecular patterns (PAMPs) and physical stressors induce high Fn14 expression in non-hematological cells resulting in a tissue injury-associated expression pattern (Winkles, 2008; Burkly et al., 2011). Accordingly, a variety of functions have been described for Fn14 in tissue repair and regeneration which, however, when excessively or chronically engaged, promote adverse effects, e.g., fibrosis and chronic inflammation (Wajant, 2013). Since tumor growth is unavoidably linked with tissue injury, Fn14 expression is regularly high in cancer cells and non-hematological cells of the tumor microenvironment (Zaitseva et al., 2022).

TNFR1, TNFR2 and Fn14 interact and are activated by ligands of the TNF superfamily (TNFSF) (Locksley et al., 2001; Bodmer et al., 2002). Ligands of the TNFSF (TNFLs) occur as type II transmembrane proteins or as soluble proteins. TNFLs are characterized by a C-terminal TNF homology domain, which promotes homotrimerization, or in a few cases, heterotrimerization of the ligand molecules, and binding to three TNFR molecules. TNFR1 and TNFR2 interact with the same set of TNFL molecules, namely, soluble TNF, membrane TNF, lymphotoxin-α (LTα) and soluble and membrane LTα_2_β heterotrimers (Bodmer et al., 2002; Kucka et al., 2021). The soluble TNF trimers (sTNF) are released from the transmembrane trimeric TNF molecules (memTNF) by proteolytic processing by the disintegrin and metalloprotease 17 (ADAM17) whereas the LΤα trimer only occurs as a secreted soluble trimer. However, LTα protomers can also form heterotrimers with the transmembrane TNFL LTβ resulting in membrane-bound but also soluble LTα_2_β and LTαβ_2_ heterotrimers (Browning et al., 1993; Browning et al., 1996). Importantly, TNFR1 and TNFR2 differ in their capability to stimulate intracellular signaling pathways upon binding to some of these ligand molecules. All ligands trigger TNFR1 signaling, TNFR2 signaling, however, is only engaged by the membrane-bound ligands (memTNF, mem LTα_2_β) despite also binding the soluble ligand molecules (sTNF, LTα, sLTα_2_β) with high affinity (Grell et al., 1995; Grell et al., 1998; Kucka et al., 2021). Since sTNF and LTα as well as memTNF and memLTα_2_β do not differ with regard to their TNFR1 or TNFR2 stimulatory properties, we will only discuss in this review the much better investigated interactions of the TNF variants with the two TNF receptors. Fn14 interacts with transmembrane TNF-like weak inducer of apoptosis (TWEAK), again a typical ligand of the TNFSF, and soluble TWEAK (sTWEAK), which originates by intracellular furin protease-mediated proteolytic processing of memTWEAK (Brown et al., 2010). Similar to TNFR2, Fn14 also differently respond to soluble and membrane-bound ligand trimers. Some Fn14-associated signaling pathways, e.g., the alternative NFκB pathway, were efficiently activated by sTWEAK and memTWEAK, while other pathways, e.g., the classical NFκB pathway, were primarily activated by memTWEAK (Roos et al., 2010).

TNF is expressed by a variety of cell types in response to a broad range of PAMPs and inflammatory cytokines, particularly in myeloid cells, T- and B cells and fibroblasts (Falvo et al., 2010). Similarly, TWEAK expression has been detected in a variety of cell types by immunohistochemistry and RT-PCR. MemTWEAK, however, has only been reported unquestionably for monocytes, dendritic cells, NK cells and T cells and a very few tumor cell lines especially after interferon-γ stimulation (Nakayama et al., 2000; Kaplan et al., 2002; Kawakita et al., 2004; Maecker et al., 2005). Thus, TNF and TWEAK seem to be coexpressed by activated myeloid cells and T cells suggesting that costimulation of TNFR1, TNFR2 and Fn14 is rather the rule than the exception in cells in the neighborhood of these cell types. Indeed, in a variety of disease models both, TNF and TWEAK, have been identified as pathology promoting molecules (Table 1).

**TABLE 1:** TNF/TNFR1-and TWEAK/Fn14-driven diseases.

Disease	Type of evidence	Refs
Acute lung injury	Fn14 blocking antibodies	Clark et al. (2000), Song et al. (2001), Zou et al. (2018)
	TNF- and TNFR1 KO mice	
	TNFR2ed-Fc treated mice	
Aortic abdominal aneurysm	Fn14-, TWEAK- and TNF KO mice sTNF inhibitor (XPro1595)-treated mice	Xiong et al. (2009), Tarín et al. (2014), Griepke et al. (2022)
Atherosclerosis	TWEAK- and TNF KO mice	Brånén et al. (2004), Muñoz-García et al. (2009), Sastre et al. (2014), Chen et al. (2015)
	TNFR1ed-Fc- and anti-TWEAK antibody-treated mice	
Atopic dermatitis/psoriasis	TNFR1- and Fn14 KO mice	Kumari et al. (2013), Peng et al. (2018)
CCl4-induced liver damage	TWEAK-, Fn14-, TNF- and TNFR1 KO mice	Simeonova et al. (2001), Sudo et al. (2005), Karaca et al. (2015), Wilhelm et al. (2016)
CIA	TNFR1 KO mice, TNFR1ed-Fc- anti-TWEAK antibody-treated mice	Mori et al. (1996), Kamata et al. (2006), Perper et al. (2006)
EAE	sTWEAK transgenic mice	Eugster et al. (1999), Desplat-Jégo et al. (2002), Desplat-Jégo et al. (2005), Mueller et al. (2005), Batoulis et al. (2014)
	Vaccination of mice with sTWEAK or Fn14ed anti-TWEAK antibody-treated mice	
	TNF- and TNFR1 KO mice anti-TNF antibody-treated mice	
γ-irradiation–induced IEC apoptosis	Fn14- and TNFR1 KO mice	Inagaki-Ohara et al. (2001), Dohi et al. (2009)
	TNFR1ed-Fc- and anti-TWEAK antibody-treated mice	
GvHD	Antagonistic anti-Fn14 antibody-treated mice	Speiser et al. (1997), Chopra et al. (2015)
	TNFR1 KO mice	
I/R renal	Anti-Fn14 antibody-treated mice	Liu et al. (2009), Hotta et al. (2011), Di Paola et al. (2013)
	TNFR1 knockout mice	
Kidney injury	TWEAK-, Fn14-, TNF- and TNFR1 KO mice	Knotek et al. (2001), Cunningham et al. (2002), Sanz et al. (2008), Ucero et al. (2013), Martin-Sanchez et al. (2018)
Induced by UUO, LPS or folic acid		
TNBS-induced colitis	TWEAK-, Fn14-, TNF- and TNFR1 KO mice	Neurath et al. (1997), Armstrong et al. (2001), Nakai et al. (2005), Dohi et al. (2009)
	Anti-TWEAK antibody- and anti-TNF antibody-treated mice	
MRL/lpr	TNFR1- and Fn14 KO mice	Deng et al. (2010a), Deng et al. (2010b), Wen et al. (2015)
	TNFR1-PLAD treated mice	
Neuropathic pain	Microinjection of Fn14 shRNA expressing AAV5	Dellarole et al. (2014), Huang et al. (2019)
	TNFR1 KO mice	
Polycystic kidney disease	TNFR2ed-Fc treated Pkd2 (+/−) mice	Li et al. (2008), Cordido et al. (2021)
	Anti-TWEAK antibody-treated conditional PKD1 KO mice	
Pressure overload	TNF- and Fn14 KO mice	Sun et al. (2007), Novoyatleva et al. (2013a), Novoyatleva et al. (2013b)

## 2 Cytotoxic TNF signaling

The death receptor subgroup of the TNFRSF can further be subdivided into three categories depending on whether and how these receptors use the DD-containing adapter protein FADD and the FADD-interacting caspase-8 molecule to induce apoptosis (Siegmund et al., 2017). CD95 (Fas, Apo1) and the two death receptors of TNF-related apoptosis inducing ligand (TRAIL), death receptor 4 (DR4, TRAILR1) and DR5 (TRAILR2), directly interact with FADD which in turn recruits procaspase-8 into the plasma membrane-associated receptor signaling complexes of these DRs, the so-called death-inducing signaling complex (DISC). Within the DISC, DR-bound FADD molecules form a filamentous “cap” serving as a nucleation core for the formation of filaments by the tandem death effector domain (tDED) of procaspase-8. This enables anti-parallel dimerization of the caspase homology domains of neighboring procaspase-8 molecules resulting in their autoproteolytic maturation, release of caspase-8 heterotetramers and eventually apoptosis induction in vulnerable cells (Fu et al., 2016; Fox et al., 2021).

TNFR1 and its close relative DR3 are also able to promote FADD-dependent maturation of procaspase-8 but do this in a different manner to the CD95-type DRs. TNFR1 and DR3 do not directly bind FADD but instead recruit the DD-containing signaling proteins TRADD and RIPK1. By yet poorly investigated mechanisms, TRADD and RIPK1 are released from the receptor signaling complexes of TNFR1 and DR3 in a way that empowers these molecules to form cytosolic complexes with FADD and procasase-8 in which autoproteolytic processing of procaspase-8 to mature caspase-8 heterotetramers become again possible. Although not fully consistently defined in the literature, two different TNFR1-induced cytosolic caspase-8 activating complexes, complex IIa and complex IIb, are distinguished dependent on their core components (TRADD, FADD, caspase-8 *versus* RIPK1, FADD, caspase-8), regulation by linear ubiquitination and relevance of RIPK1’s kinase activity (Dondelinger et al., 2016; Griewahn et al., 2019; Kondylis and Pasparakis, 2019; van Loo and Bertrand, 2023). In particular, both complexes are subject to regulation by FLIP proteins and TRAF2 and the cIAPs. Since TRADD and RIPK1 have a strong intrinsic tendency to autoaggregate, it is tempting to speculate that FADD-instructed formation of procaspase-8 filaments becomes also relevant in the TNFR1-induced mode of caspase-8 activation. The direct *versus* and indirect mode of FADD-mediated caspase-8 activation by the TNFR1- and CD95-type DRs is also reflected by the kinetics of caspase-8 activation. Assembly of the CD95 DISC and caspase-8 activation becomes visible within few minutes while TNFR1-induced complex IIa/b formation and caspase-8 processing is much slower and takes hours (Scaffidi et al., 1998; Micheau and Tschopp, 2003).

The receptors of a third heterogeneous category of DRs induce no apoptosis or do this only by poorly understood mechanisms without evidence for a role of FADD and caspase-8. The three subcategories of the DR group also differ with respect to necroptosis induction. The CD95-type DRs as well as TNFR1 and DR3 are able to induce necroptosis. Central step is here the formation of a DR-induced cytosolic complex, called ripoptosome or necrosome, in which autocatalytically activated dimers of RIPK1 bind and activate RIPK3, which in turn phosphorylates the mixed lineage kinase domain-like (MLKL) pseudokinase triggering conformational changes in the latter resulting in translocation to the plasmamembrane, pore formation and cell lysis (Dondelinger et al., 2016; Griewahn et al., 2019; Kondylis and Pasparakis, 2019; van Loo and Bertrand, 2023). Worth mentioning, the DR-induced interaction of activated RIPK1 and RIPK3 results in amyloid-like structured complexes (Mompeán et al., 2018). How formation of the necrosome is engaged at the molecular level, however, is again fundamentally different between CD95-type DRs and TNFR1 with respect to the role of FADD. Deficiency of FADD and also FADD/TRADD double-deficiency do not affect and even potentiate TNFR1-induced necroptosis whereas lack of FADD abrogates CD95-type DR-induced necroptosis (Holler et al., 2000; Füllsack et al., 2019). As FADD and FADD + TRADD can be dispensable for necroptosis, the necrosome can obviously form independently from the apoptotic complexes IIa and IIb but this does not rule out that the necrosome can also secondarily originate from RIPK1-containing complexes II.

**FIGURE 2:**
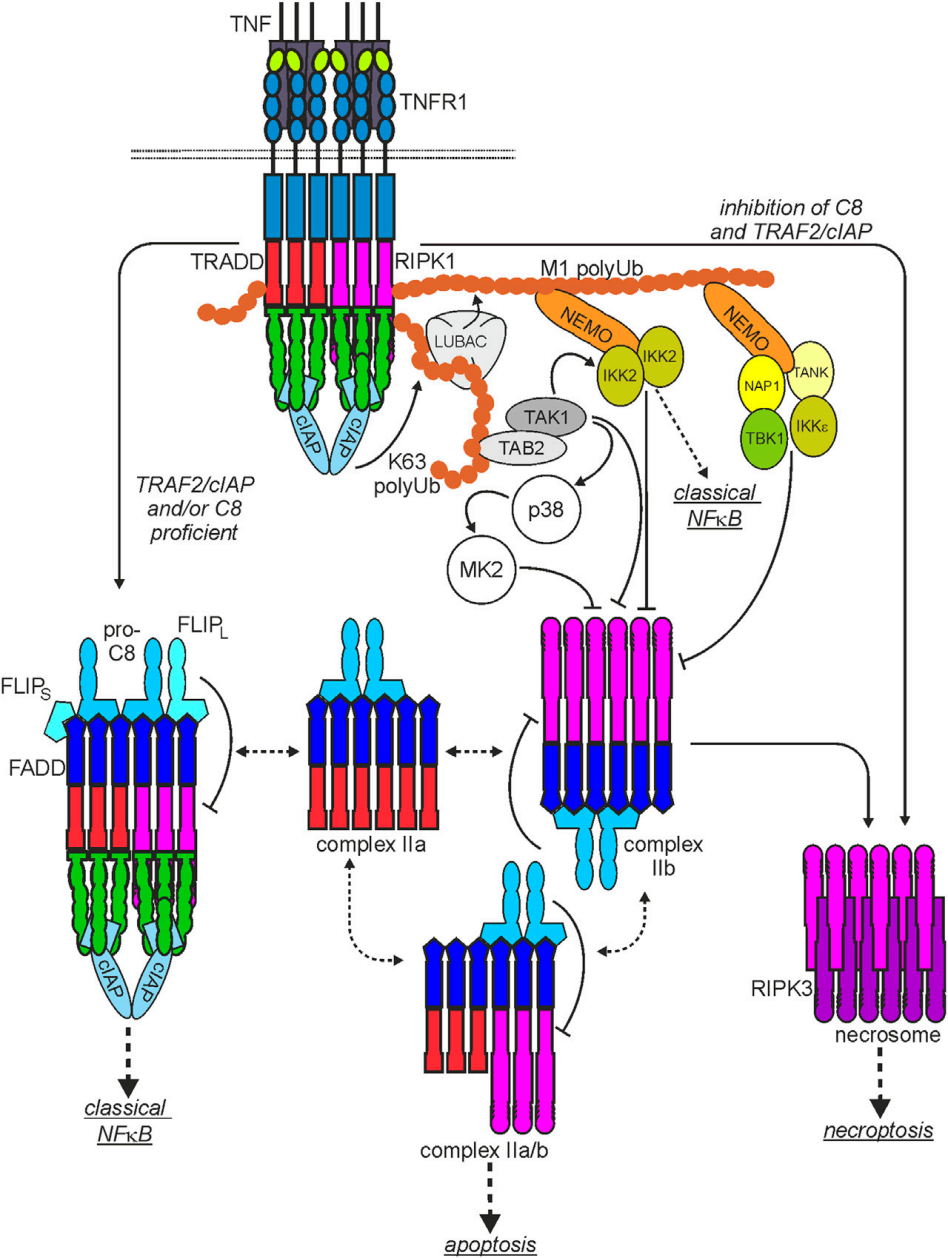
Scheme of the major TNF-induced TNFR1-instructed signaling complexes. The plasma membrane-associated TNFR1-containing signaling complex I is rapidly formed. After TNFR1 internalization TRADD and/or RIPK1 dissociate from complex I and become part of various cytoplasmic signaling complexes with FADD, procaspase-8 (complex IIa, IIb, IIa/b) or RIPK3 (necrosome) with the capability to trigger cell death. Please note: I) There is good evidence that two or more liganded TNFR1 trimers are required for complex I formation but it is currently not clear whether this “oligomeric” requirement is maintained for the secondarily formed cyroplasmic complexes. II) It is not clear whether and if yes to which extend the various complexes exchange components. III) Shown are the functional relevant core components of the various cytosolic complexes. Thus, these complexes can still be decorated by regulatory components, especially by interaction with TRAF2. For more details, please see main text.

As mentioned above, in response to ligand-binding, TNFR1 recruits TRADD and RIPK1 by homotypic interactions of the DDs contained in these molecules. There is evidence that TRADD and RIPK1 compete for TNFR1 binding and that each of the two cytosolic DD-containing proteins is independently able to mediate TNFR1-induced apoptosis (Figure 2). Although TRADD and RIPK1 can bind and activate the FADD-caspase-8 dyad in the TNFR1-induced cytosolic complexes IIa and IIb (Figure 2), FADD and caspase-8 are not recruited to the plasma membrane-associated TNFR1 signaling complex (Micheau and Tschopp, 2003). Since TRADD and RIPK1 interact with FADD this opens the possibility that TNFR1 and FADD compete for binding to these proteins (Varfolomeev et al., 1996). Importantly, recruitment of TRADD and RIPK1 to TNFR1 (or DR3) is not only an obligate step preceding TNFR1-induced formation of the complex II variants and the necrosome but also instruct the formation of a TNFR1-associated multiprotein signaling complex at the plasma membrane, called complex I (Micheau and Tschopp, 2003; Dondelinger et al., 2016; Kondylis and Pasparakis, 2019; van Loo and Bertrand, 2023). Both proteins are independently able to recruit secondarily TNF receptor associated factor-2 (TRAF2), a scaffold protein with E3 ligase activity, to the TNFR1 signaling complex along with the TRAF2-interacting E3 ligases cellular inhibitor of apoptosis-1 (cIAP1) and cIAP2 (cIAPs). The latter strongly K63-ubiquitinate RIPK1 but also some other components of the TNFR1 signaling complex (Figure 2). This creates binding sites for the TGFβ activating kinase-1 (TAK1)-associated binding protein (TAB)-TAK1 complex and the linear ubiquitin chain assembly complex (LUBAC). The LUBAC modifies again RIPK1 but also TRADD and TNFR1 with ubiquitin, this time with M1-linked chains (Dondelinger et al., 2016). The linear polyubiquitin chains in the TNFR1 signaling complex enable then the recruitment of the IκBα-kinase (IKK) complex composed of the ubiquitin-binding scaffold protein NEMO and IKK1 and IKK2 and the NEMO-NAP1-TANK-TBK1-IKKε complex (Lafont et al., 2018; Xu et al., 2018). The latter phosphorylates and thereby inhibits RIPK1-mediated cell death, largely without affecting TNF-induced NFκB signaling (Lafont et al., 2018; Xu et al., 2018). Recruitment of the IKK complex, however, enables TAK1 to activate the IKK2 subunit of the IKK complex triggering the key events of the classical NFκB pathway, namely, phosphorylation and K48 ubiquitination of IκBα, proteasomal IκBα degradation and release of IκBα-blocked NFκB transcription factor dimers. TRADD, RIPK1, TRAF2 and TAK1 are not only of central relevance for TNFR1-induced NFκB signaling and cell death induction but also enable TNFR1 to stimulate the MAPK p38 and cJun N-terminal kinase (JNK) signaling cascades (Totzke et al., 2020). In addition to the yet mentioned factors several other proteins have been identified as components of the TNFR1 signaling complex which modulate the activity of one or more of the TNFR1-associated signaling pathways and thus the net effect of TNFR1 activation.

Similar to TNFR1, the CD95-type DRs also stimulate the classical NFκB pathway and various MAPK cascades (Siegmund et al., 2017) under crucial involvement of TRADD and RIPK1 but this issue has been limitedly investigated, yet, with complex results. For example, early, thus cell death-independent, JNK signaling is abrogated in TRAIL-stimulated TRAF2-deficient murine embryonal fibroblasts while NFκB activation remains unaffected (Lin et al., 2000; Varfolomeev et al., 2005). In contrast, in the murine A20 cell line and in human HCT116 and HT1080 cells, TRAF2-deficiency significantly reduced TRAIL-DR-induced activation of the classical NFκB signaling pathway (Zhang et al., 2015; Kreckel et al., 2019). Intriguingly, in contrast to TNFR1, the CD95-type DRs require for classical NFκB signaling also FADD and caspase-8 (Siegmund et al., 2017). There is evidence that TRAF2, RIPK1 and the LUBAC also recruit to CD95-type DRs, however, these recruitment processes are experimentally more difficult to demonstrate than the recruitment to TNFR1 suggesting that recruitment of TRAF2, RIPK and LUBAC to CD95-type DRs is less efficient or results in less stable complexes than in the case of TNFR1 (Siegmund et al., 2017).

Induction of apoptosis by TNFR1 and other death receptors can also occur by indirect activation of effector caspases via the generation of reactive oxygen species (ROS) and triggering of prolonged JNK activation under crucial involvement of the MAP3K apoptosis signal-regulating kinase 1 (ASK1) and/or mammalian Ste20-like kinase 1 (MST1) (Nishitoh et al., 1998; Hoeflich et al., 1999; Roh and Choi, 2016). A major molecular link between ROS formation and TRAF2-mediated ASK1 activation is thioredoxin (Trx). ROS production in response to TNF triggers dissociation of ASK1-Trx complexes, which sequester ASK1 in an inactive state, enabling TRAF2 to associate and stimulate ASK1 (Liu et al., 2000). Likewise, ROS also trigger dissociation of Trx-MST1 complexes containing inactive MST1 (Roh and Choi, 2016). Another mechanism contributing to TNF-induced ROS- and ASK1/MST1-mediated sustained JNK activation and apoptosis seems to be the inhibition of MAP kinase phosphatases (Kamata et al., 2005). Worth mentioning, ROS production in response to TNFR1 engagement can occur by complex mechanisms including direct activation of NADPH oxidase by the TNFR1-interacting riboflavin kinase (Yazdanpanah et al., 2009) but also in crosstalk with mitochondrial damage occurring, e.g., in the intrinsic branch of apoptosis signaling.

## 3 Cytotoxic TNFR1 signaling is controlled at multiple levels

Despite the ubiquitous expression of TNFR1, TNF fails to induce a robust cell death response in the huge majority of cell lines and primary cells and instead triggers a wide variety of cell death-independent processes reaching from induction of gene synthesis of hundred of proteins over cell migration to proliferation and cellular differentiation. Thus, there have to exist mechanisms that hold in check the cytotoxic branch(es) of TNFR1 signaling.

### 3.1 TNFR1-induced classical NFκB signaling inhibit TNFR1-associated cell death pathways

The first mechanism that has been identified in this respect is TNFR1-induced activation of the classical NFκB pathway which results in the production of several proteins that interfere with death receptor-induced cell death signaling or protect from cell death processes more general at the effector level (Table 2).

**TABLE 2:** NFκB-regulated factors protecting from TNFR1-induced cell death.

Factor	Function	Ref
FLIP_S_	Inhibits first step in procaspase-8 maturation	Kreuz et al. (2001), Micheau et al. (2001)
FLIP_L_	Inhibits second step of procaspase-8 maturation	
cIAP1, cIAP2	K63 ubiquitination	Chu et al. (1997), Stehlik et al. (1998), Wang et al. (1998)
	Classical NFκB signaling	
	Caspase inhibition	
MnSOD	Scavenging of superoxide radicals	Wong et al. (1989), Wan et al. (1994)
Ferritin heavy chain	Sequestration of iron resulting in reduced TNF-induced ROS accumulation	Kwak et al. (1995), Pham et al. (2004)
A20^a^	Stabilizes M1 ubiquitination in TNFR1 signaling complex	Krikos et al. (1992), He and Ting (2002), Won et al. (2010), Draber et al. (2015)
	ASK1 degradation	
Bfl1	Inhibits intrinsic apoptosis pathway	Zong et al. (1999), Cheng et al. (2000)
Bcl2	Inhibits intrinsic apoptosis pathway	Catz and Johnson (2001)
BclxL	Inhibits intrinsic apoptosis pathway	Cheng et al. (2000)
xIAP	Caspase inhibition	Stehlik et al. (1998)

^a^ A20 has complex function in TNFR1 signaling and may also enhance cell death-induction under certain situations (Garcia-Carbonell et al., 2018).

The relevance of NFκB activation for the inhibition of cytotoxic signaling of TNFR1 is highlighted by the fact that deficiency in the transcription factor subunit RelA results in embryonal lethality which can be rescued by knockout of the TNF or TNFR1 gene (Doi et al., 1999; Rosenfeld et al., 2000). An important aspect is here, that TNFR1-induced NFκB activation is significantly faster than the formation of complex IIa/b and caspase-8 activation. The relevance of the TNF-inducible, but also constitutive expression of cell death-inhibitory proteins is eventually mirrored by the fact that protein synthesis inhibitors, e.g., cycloheximide, or mRNA transcription inhibitors, e.g., actinomycin D, are often used in cell death research to sensitize for DR-, especially TNFR1-induced cell killing. Worth mentioning, some of the NFκB-regulated protective factors can also be expressed without TNFR1 engagement and adjust the cellular sensitivity for TNFR1- and DR-induced cell killing.

### 3.2 Control of TNFR1-induced cell death signaling by FLIP proteins and caspase-8

The most powerful and complex regulators of TNFR1- and CD95 type-induced cell killing seem to be caspase-8 and the protein isoforms of the cellular FLICE-like inhibitory protein (cFLIP) gene. There are three protein isoforms of FLIP: FLIP-L, FLIP-S and FLIP-R, which all interact with FADD and which all form heterodimers with procaspase-8 (Nakano et al., 2017; Ivanisenko et al., 2022). The mentioning of caspase-8 here as an inhibitory cell death regulator may appear surprising but reflects the fact that the processing of the different substrates of caspase-8 can result in apoptosis and/or pyroptosis induction but also in inhibition of necroptosis and NFκB signaling. For example, cleavage of effector caspases and Bid results in the generation of apoptosis promoting peptides while gasdermin D cleavage can lead to pyroptosis, and processing of RIPK1 suppresses necroptotic and NFκB signaling (Schwarzer et al., 2020; Han et al., 2021). Thus, caspase-8 activation in context of TNFR1 signaling is not only required for apoptosis induction but also controls the balance between various TNFR1 signaling pathways. Intriguingly, the different FLIP protein isoform can modify the cell death and regulatory effects of caspase-8 in DR signaling. FLIP-S and FLIP-R differ in their C-terminal tail and share with FLIP-L and procaspase-8 a N-terminal tandem DED. FLIP-S and FLIP-R interact with the tDED of procaspase-8 and become incorporated into the CD95 DR type-induced DISC and prevents formation of cytotoxic filaments and initial cleavage of procaspase-8 to p41/p43 and p10 (Nakano et al., 2017; Ivanisenko et al., 2022). In contrast, FLIP-L resembles procaspase-8 and comprises in addition to the N-terminal tDED also a caspase homology domain. FLIP-L forms heterodimers with procaspase-8 which undergoes a first initial processing step but are then arrested at this intermediate maturation state resulting in heterotetramers composed of caspase-8 p42/43 and p10 and the corresponding FLIP-L processing intermediates which are not released in the cytoplasm. (Nakano et al., 2017; Ivanisenko et al., 2022). As a consequence, despite promoting DR-induced caspase-8 activation to some extent, FLIP-L expression inhibits DR-induced apoptosis. The caspase-8-FLIP-L intermediates cleave, however, a limited set of substrates involving RIPK1, RIPK3 and Cyld and thereby inhibits necroptotic signaling (Schwarzer et al., 2020; Han et al., 2021; Ivanisenko et al., 2022).

### 3.3 Regulation of cytotoxic TNFR1 signaling by TRAF2 and cIAPs

Early on after the identification of TRAF2 and the cIAPs as components of the signaling complexes of TNFR1 and TNFR2, there was first evidence that TRAF2 and the cIAPs limit TNFR1-induced caspase-8 activation and apoptosis. The concomitant expression of TRAF2, TRAF1 and the two cIAPs was found to inhibit TNF-induced caspase-8 activation (Wang et al., 1998). In CD4^+^CD8^+^ double-positive thymocytes of TRAF2-deficient mice, TNF- but not anti-CD95 antibody-induced cell death was found to be enhanced and embryonal fibroblasts derived of TRAF2-defcient mice were also sensitized for TNF in the presence of cycloheximide (Yeh et al., 1997). Worth mentioning, TNF-induced NFκB signaling remained largely unaffected in TRAF2-deficient mice arguing for a NFκB-independent cytoprotective activity of TRAF2 (Hoeflich et al., 1999). Likewise, studies with cIAP antagonists (= SMAC mimetics) and cIAP1- and/or cIAP2-deficient mice revealed a significant sensitization of various cell types for TNF-induced apoptosis (Vince et al., 2007; Wang et al., 2008; Moulin et al., 2012). Moreover, the antiapoptotic activity of the cIAPs in TNF signaling was found to be TRAF2-dependent (Vince et al., 2009). Indeed, several of the molecules and protein complexes discussed above which recruit to the TNFR1 signaling complex in a TRAF2- and/or cIAP-dependent manner have been found to antagonize TNF-induced cell death signaling independent from NFκB signaling (Meng et al., 2021; van Loo and Bertrand, 2023). For example, the TAB-TAK1 complex phosphorylates human RIPK1 on serine 320 and thereby inhibits RIPK1-dependent apoptosis independently from NFκB signaling and this was found to be severely reduced in the absence of cIAPs (Figure 2) (Geng et al., 2017). TAK1 activation also enables p38-mediated activation of MK2 which also directly phosphorylates human RIPK1 on serine 320 and serine 335 in the cytosol antagonizing complex IIb formation (Figure 2) (Dondelinger et al., 2017; Jaco et al., 2017; Menon et al., 2017). Furthermore, the LUBAC which M1 ubiquitinates RIPK1 and controls TNF-induced cell death in a complex manner beyond classical NFκB signaling largely fails to recruit to TNFR1 in TRAF2-deficient and cIAP1/2-double deficient cells (Haas et al., 2009). In a similar fashion, the IKK complex, which is recruited to the TNFR1 signaling complex in a TRAF2-dependent manner, phosphorylates RIPK1 on serine 25 and inhibits TNF-induced RIPK1-mediated apoptosis (Devin et al., 2000; Dondelinger et al., 2015).

### 3.4 Regulation of cytotoxic TNFR1 signaling by TRAF2 interacting TNFRs

It was found that recruitment of TRAF2 by TNFR2 correlated with enhanced TNFR1-induced caspase-8 activation and that overexpression of TRAF2 and/or the cIAPs, furthermore, confers resistance against TNFR1-induced apoptosis (Duckett and Thompson, 1997; Weiss et al., 1997; Declercq et al., 1998; Wang et al., 1998; Chan and Lenardo, 2000). TNFR2-induced recruitment of TRAF2 and the cIAPs is typically followed by cIAP1-mediated K48 ubiquitination of TRAF2 and proteasomal degradation of TRAF2 (Li et al., 2002; Wu et al., 2005). However, the effects of TNFR2 on TNFR1 signaling are more rapidly evident than TRAF2 degradation suggesting that depletion of the cytosolic pool of TRAF2 by relocation of TRAF2 molecules to TNFR2 is already sufficient to tip the balance of TNFR1 signaling towards cell death induction by limiting the availability of TRAF2 and the cIAPs (Fotin-Mleczek et al., 2002; Wicovsky et al., 2009a).

Intriguingly, caspase-8 activation and apoptosis induction by CD95 and the TRAIL DRs are not enhanced by TNFR2-dependent TRAF2 depletion what is in good accordance with the positioning of TRAF2 and cIAPs *versus* the FADD-caspase-8 dyad in the signaling network engaged by these two subgroups of DRs (Weiss et al., 1998; Fotin-Mleczek et al., 2002). While in TNFR1 signaling TRAF2 and the cIAPs act upstream of FADD and caspase-8, these molecules act with a reciprocal hierarchy in CD95-and TRAIL DR signaling (Siegmund et al., 2017).

At the beginning, the antiapoptotic role of TRAF2 in TNFR1 signaling was attributed to its contribution to TNFR1-induced classical NFκB activation (Kucharczak et al., 2003). A closer look on the kinetics of the TNFR1- and TNFR2-associated signaling processes involving TRAF2 and the cIAPs, however, suggests that these molecules protect against TNFR1-induced cell death also independent from their role in NFκB signaling. The depletion of cytosolic TRAF2 and the cIAPs needs 1–3 h to elicit functional consequences while the NFκB-inducing TNFR1 signaling complex is formed within the minute range (Shu et al., 1996; Fotin-Mleczek et al., 2002; Wicovsky et al., 2009a). Accordingly, the latter and NFκB signaling remain unaffected when TNFR1 and TNFR2 are costimulated although there is enhanced TNFR1-induced caspase-8 activation and apoptosis (Fotin-Mleczek et al., 2002). However, when TNFR2 is stimulated several hours prior TNFR1, there is not only enhanced TNFR1-dependent apoptosis signaling but also reduced TNFR1-induced classical NFκB activation. Worth mentioning, the effects of TNFR2 priming on TNFR1 signaling are highly reminiscent to that of TRAF2- or cIAP1/2-deficiency (Table 3).

**TABLE 3:** Comparison of the effects of TNFR2 or Fn14 priming and TRAF2- and cIAP1/2-deficiency on TNF signaling.

TNFR1 signaling event	TRAF2 deficiency	cIAP1/2 deficiency	TNFR2/Fn14 priming	Ref
TRADD/RIPK1 recruitment	Largely unchanged	Largely unchanged	Unaffected	Devin et al. (2000), Wicovsky et al. (2009a), Wicovsky et al. (2009b), Varfolomeev et al. (2012)
RIPK1 ubiquitination	Strongly reduced	Strongly reduced	Strongly reduced	Mahoney et al. (2008), Wicovsky et al. (2009a), Wicovsky et al. (2009b), Varfolomeev et al. (2012)
IKK recruitment	Inhibited	Inhibited	Strongly reduced	Devin et al. (2000), Wicovsky et al. (2009a), Wicovsky et al. (2009b)
Caspase-8 activation	Enhanced	Enhanced	Enhanced	Wicovsky et al. (2009a), Wicovsky et al. (2009b), Moulin et al. (2012), Kreckel et al. (2019)
Apoptosis induction	Enhanced	Strongly reduced	Enhanced	Mahoney et al. (2008), Wicovsky et al. (2009a), Wicovsky et al. (2009b), Petersen et al. (2015)
Necroptosis induction	Enhanced	Enhanced	Enhanced	Wicovsky et al. (2009b), McComb et al. (2012), Moulin et al. (2012), Karl et al. (2014), Petersen et al. (2015)
Early JNK activation	Strongly reduced	Inhibited	Inhibited	Yeh et al. (1997), Weiss et al. (1998), Wicovsky et al. (2009a), Wicovsky et al. (2009b), Varfolomeev et al. (2012)
Classical NFκB activity	Mildly reduced	Attenuated	Attenuated	Yeh et al. (1997), Fotin-Mleczek et al. (2002), Wicovsky et al. (2009a), Wicovsky et al. (2009b), Varfolomeev et al. (2012)
TNFR2 signaling event				
NIK accumulation	Enhanced	Enhanced	Enhanced	Vallabhapurapu et al. (2008), Zarnegar et al. (2008), Roos et al. (2010)
p100 processing	Enhanced	Enhanced	Enhanced	Saitoh et al. (2003), Varfolomeev et al. (2007), Vince et al. (2007), Vallabhapurapu et al. (2008), Rauert et al. (2010)

IAP antagonists resulting in the autocatalytic degradation of cIAPs and TRAF2 depletion by engagement of TRAF2-interacting TNFRs have largely similar sensitizing effects on TNFR1-induced cell death. This suggests that the survival effect of TRAF2 in context of TNFR1 signaling is mainly due to its ability to recruit the cIAPs to the TNFR1 signaling complex (Vince et al., 2009) and possibly to the cytosolic complexes IIa and IIb in order to interfere with caspase-8 activation. The molecular mechanism(s) by which TRAF2 and the cIAPs antagonizes TNFR1-induced apoptotic caspase-8 activity independently form inhibiting NFκB-dependent expression of survival proteins are complex and only initially understood. One aspect that could play a role here is K48-ubiquitination of the p43 and p18 fragment of caspase-8 by TRAF2 which stimulates the proteasomal degradation of theses peptides, a process that has been observed in context of TRAIL-induced apoptosis where it limits cell killing (Gonzalvez et al., 2012; Xu et al., 2017). A role of TRAF2-dependent degradation of intermediates of caspase-8 maturation in the apoptotic crosstalk of TNFR1 and TNFR2 appears at the first glance in contradiction to the aforementioned observation that TNFR2 selectively enhances TNFR1- but not TRAIL- or CD95L-induced apoptosis. However, this “contradiction” may resolve again after a closer look on the kinetics of the processes involved. Caspase-8 maturation in the DISC of CD95-type DRs occurs within very few minutes making it unlikely that the slowly occurring TNFR2-induced TRAF2 depletion gains relevance for DISC-associated caspase-8 activation (Scaffidi et al., 1998). In contrast, the time course of caspase-8 activation in the cytosolic complexes IIa and IIb is rather slow (Micheau and Tschopp, 2003) so that the likewise slow process of TNFR2-mediated TRAF2 depletion can gain relevance. Indeed, TNF- and TRAIL-induced caspase-8 activity is reduced in TRAF2-deficient HCT116 cells (Kreckel et al., 2019). Interestingly, K48-ubiquitination of the caspase-8 fragments seems to be directly mediated by TRAF2 itself via its N-terminal RING domain and not by the TRAF2-interacting cIAPs (Gonzalvez et al., 2012). An authentic E3 ligase activity of TRAF2 is, however, questioned by structural data and *in vitro* results showing that TRAF2 is unable to interact with E2 conjugating enzymes (Yin et al., 2009). A possible resolution of these inconsistent findings could lie in a study describing sphingosine-1-phosphate (S1P) as a cofactor of TRAF2 required by the latter to K63-ubiquitinate RIPK1 in the presence of E2 conjugating enzymes (Alvarez et al., 2010). Unfortunately, however, the role of S1P for TRAF2-mediated K48-ubiquitination of caspase-8 has not been investigated yet and Alvarez et al. found no evidence for K48-ubiquitination by TRAF2 in the presence of E2 molecules. Moreover, studies with sphingosine kinase 1 (Sphk1)-deficient cells and mice argue against a major role of this S1P generating enzyme in TRAF2/RIPK1-related functions in TNF signaling and found, in contrast to Alvarez et al., no reduced TNF-induced NFκB signaling in murine embryonal fibroblasts of Sphk1 knockout mice (Etemadi et al., 2015). In sum, there is certainly a need for additional studies to finally and fairly comment on the relevance of TRAF2 as an authentic E3 ligase in TNF signaling. The TRAF2-associated cIAP E3 ligases, however, have clear relevance in concert with TRAF2 when RIPK1-dependent control of apoptosis is considered. In context of TNFR1 signaling, the TRAF2-associated cIAPs can modify RIPK1 with K48- or K63 polyubiquitin chains enabling proteasomal degradation of RIPK1 (K48 ubiquitination) but also the direct or indirect recruitment (K63 ubiquitination) of downstream acting molecules, such as the TAB-TAK1 complex, the IKK complex, the NEMO-NAP1-TANK-TBK1-IKKε complex and the LUBAC (Dondelinger et al., 2016). Consequently, TRAF2 and the cIAPs have the potential to affect all the TNFR1-related functions of these molecules reaching from NFκB activation over inhibition of RIPK1-dependent apoptosis to control of RIPK1-mediated necroptosis.

Indeed, TNFR2 and TRAF2 depletion not only enhances TNFR1-induced apoptosis but also TNFR1-induced necroptosis (Siegmund et al., 2016). Likewise, TRAF2-deficient cells showed enhanced sensitivity for DR-induced necroptosis (Karl et al., 2014; Petersen et al., 2015). In view of TRAF2’s caspase-8 inhibitory activity, its additional necroptosis inhibitory activity is surprising because caspase-8 activation inhibits necroptotic TNFR1 signaling by cleavage of RIPK1 and other substrates, such as RIPK3 and Cyld (Orning and Lien, 2021). Accordingly, TNFR1-induced necroptosis typically is only observable when caspase-8 activation is compromised, for example, naturally by genetic alterations in tumor cells (Teitz et al., 2001) or pathogen-encoded factors (Mocarski et al., 2011), such as CrmA or vICA, or artificially by use of drugs or genetic engineering.

In accordance with a central role of the availability of TRAF2-cIAP1/2 complexes for the quality of TNFR1 signaling, it has been observed that other TNFRs with the ability to recruit TRAF2, e.g., Fn14, CD30 and CD40, also sensitize for TNFR1-induced apoptosis. Especially, enhancement of TNFR1-inducd apoptosis by Fn14 has been studied in more detail and revealed several similarities with the apoptotic TNFR1-TNFR2 crosstalk. Thus, Fn14 preferentially enhances again TNFR1-induced apoptosis and shows no effect on apoptosis induction by the CD95-type death receptors and when stimulated prior TNFR1, Fn14 engagement again comes along with inhibition of TNFR1-indued NFκB activation (Wicovsky et al., 2009b). Moreover, Fn14 engagement also results in enhanced necroptotic TNFR1 signaling (Wicovsky et al., 2009b). Interestingly, with respect to necroptotic signaling, Fn14 “looses” the preference/selectivity for TNFR1. Thus, Fn14 also enhances CD95-and TRAIL-induced necroptosis (Karl et al., 2014). This might reflect that DR-induced necroptosis occurs in a secondarily formed cytoplasmic complex with the ability to interact with TRAF2. Indeed, in context of TRAIL DR-induced activation of the classical NFκB pathway TRAF2 and the cIAPs along with FADD and RIPK1 have been identified as components of a common protein complex (or components of different protein complexes sharing partly the same components) (Varfolomeev et al., 2005). However, how this cytoplasmic TRAF2-containing NFκB signaling complex is related to the necroptosis-initiating RIPK1/RIPK3/MLKL-containing ripoptosome/necrosome is not fully clear. Whether the complexes compete for their components, e.g., RIPK1, or form sequentially or independently, or can convert into each other have not been addressed, yet. Stimulation of Fn14 also enhances TLR3-induced RIPK1-mediated necroptosis. Thus, depletion of TRAF2 and the cIAPs seems to generally result in enhanced necroptosis irrespective of the RIPK1-activating receptor considered (Anany et al., 2018; Martin-Sanchez et al., 2018).

## 4 The cytotoxic TNFR1-Fn14 crosstalk in the intestine

As already initially described above, the default state of TNFR1 activities does not include cytotoxic signaling and accordingly the huge majority of cells and cell types do not die in response to TNF exposure. A remarkable and clinically important exception are intestinal epithelial cells (IECs). TNF-induced TNFR1-dependent cell death of IECs is well established and has been early on demonstrated *in vivo* with recombinant TNF (Piguet et al., 1987; Inagaki-Ohara et al., 2001). More important, TNF-induced cell death of IECs has been likewise documented manifold in a variety of diseases including sepsis, graft-versus-host disease (Piguet et al., 1987), infections (Liu et al., 2006) and various murine colitis models (Neurath et al., 1997; Corazza et al., 1999; Marini et al., 2003; Roulis et al., 2011) and has also been implicated in radiation-induced p53-mediated and parenteral nutrition-induced IEC apoptosis (Inagaki-Ohara et al., 2001; Feng et al., 2015). Intriguingly, there is likewise broad and comprehensive evidence that TWEAK and Fn14 contribute to intestine-associated complications. Fn14 is upregulated in IECs during colitis and the severity of the latter is reduced in TWEAK-deficient mice (Dohi et al., 2009). TWEAK-deficient mice treated with γ-irradiation, furthermore, show reduced crypt progenitor cell apoptosis (Dohi et al., 2009). Intriguingly, the intestine damage-inducing cytokine IL13, but also TNF, induce apoptosis in intestinal explants and this is reduced in intestinal explants of TWEAK- and Fn14-deficient mice. TNF blockade, furthermore, inhibits in this model IL13-induced apoptosis without a beneficial effect of additional Fn14-deficiency. This suggests that both engagement of TNFR1 and Fn14 are required for the observed apoptotic response (Kawashima et al., 2011). Evidence for the relevance of the cytotoxic Fn14-TNFR1 crosstalk for the killing of IECs comes also from the trinitrobenzene sulfonic acid (TNBS)-induced acute colitis model (Dohi et al., 2014). In this model, both blockade of the TWEAK-Fn14 system and the TNF-TNFR1 system resulted in reduced disease activity and combined inhibition of both systems showed synergistic therapeutic activity (Dohi et al., 2014). Noteworthy, the induction of IEC apoptosis in healthy mice by treatment with recombinant TNF is significantly reduced by coapplication of an ADCC-dead Fn14-blocking antibody arguing for a general involvement of the cytotoxic Fn14-TNFR1 crosstalk in TNF/TNFR1-dependent IEC apoptosis (Chopra et al., 2015). In line with this, cIAP1-defciency which “mimic” TWEAK-induced depletion of TRAF2-cIAP1 complexes, also result in an increased apoptotic sensitivity of IECs for TNF-induced apoptosis (Grabinger et al., 2017).

Colitis is associated with an increased risk to develop colon cancer. Interestingly, in the AOM-DSS- and the DSS-induced colitis models, the rate of tumor development after recovery from colitis is increased in Fn14-deficient mice but also in TNFR1-deficient mice possibly reflecting insufficient TNFR1/Fn14-mediated IEC apoptosis leading to uncontrolled compensatory proliferation and/or outgrowth of yet less apoptosis-sensitive precancerous cells eventually resulting in cancer development (Popivanova et al., 2008; Di Martino et al., 2016).

The relevance of keeping in check the cytotoxic Fn14-TNFR1-crosstalk is also in accordance with observations derived of genetically engineered mice. RIPK1 deletion in the intestinal epithelium is associated with apoptosis of IECs but can be largely rescued by crossing with TNFR1-deficient mice (Takahashi et al., 2014). Furthermore, FADD deletion in IECs results in RIPK3-dependent necroptosis of IECs and colitis which are strongly reduced in TNF- or TNFR1-deficient mice (Welz et al., 2011; Dannappel et al., 2014). Likewise, IEC-specific cFLIP-deficient mice, which die perinatal, were partly rescued by crossing with TNFR1-deficient mice (Piao X. et al., 2012). Worth mentioning, TNF-mediated cell death of IECs in these genetic models is typically also ameliorated by antibiotics or MyD88-deficiency suggesting that TNF triggers a feed-forward cycle in the intestine of IEC cell death, causing increased epithelial barrier permeability and entry of bacteria, along with TNF induction by the latter eventually resulting in severe tissue destruction (Takahashi et al., 2014). It is tempting to speculate that Fn14 engagement also contributes to this vicious cycle as it is strongly induced by LPS in IECs (Qi et al., 2017). Moreover, mice deficient for TRAF2, the major TNFR1-Fn14 signaling connecting knot (Table 3), display severe colitis and increased apoptosis of IECs which both can be largely rescued by crossing with TNFR1-deficient mice (Piao et al., 2011; Piao J. H. et al., 2012).

Sequestration of TRAF2 and the cIAPs by Fn14 or TNFR2 could also be of relevance in cell death occurring during acute kidney injury or in pathogen-induced hyperinflammation in patients with X-linked inhibitor of apoptosis (XIAP)-deficiency triggered by TLR- and TNF- and TNFR2-mediated myeloid cell death (Lawlor et al., 2017; Martin-Sanchez et al., 2018).

## 5 The cytotoxic TNFR1-Fn14 crosstalk in cancer immune checkpoint blockade therapy

**FIGURE 3:**
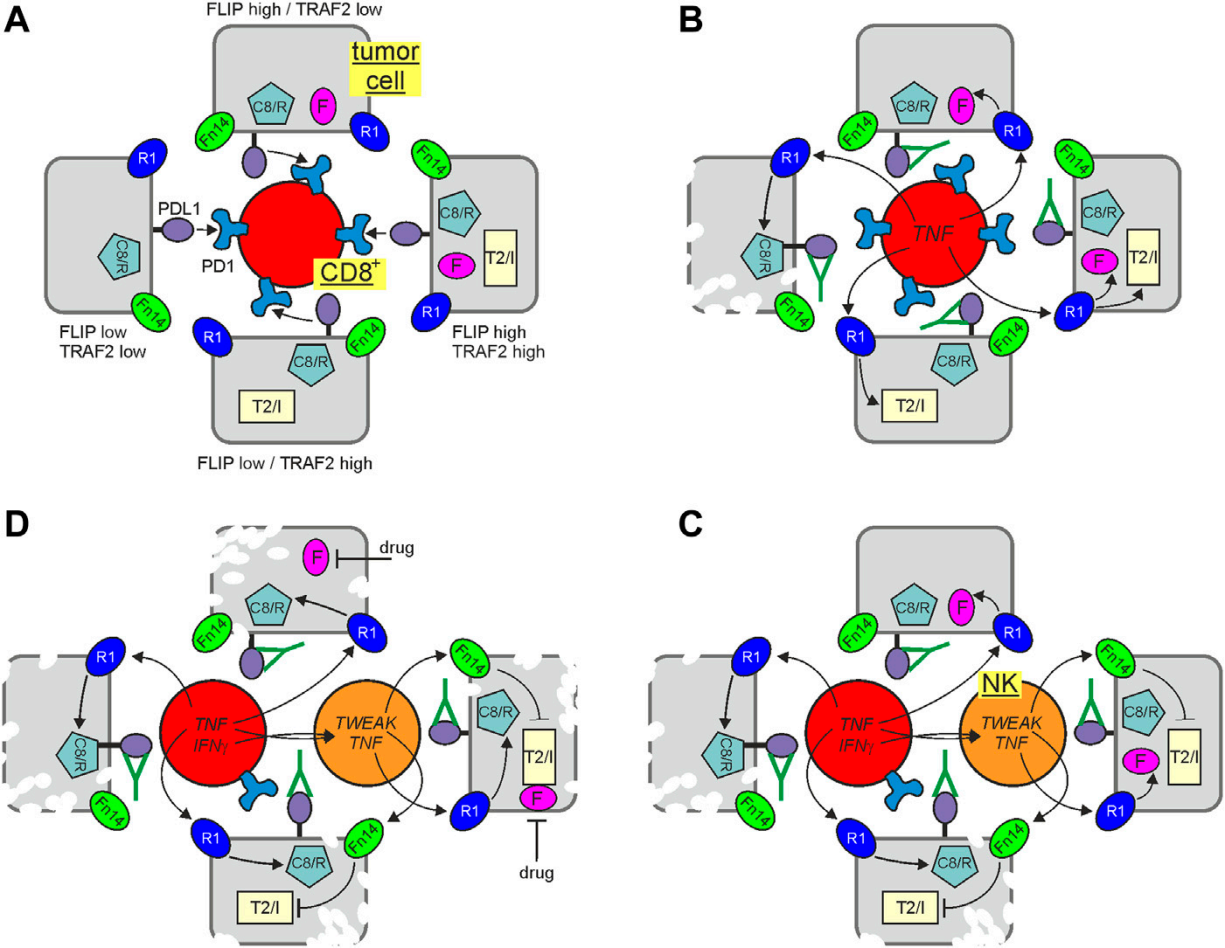
Immune check point blockade (ICB) and cytotoxic TNFR1 signaling. Tumor cells may differ in their potential to control TNFR1-induced cell death signaling due to varying levels of FLIP proteins and TRAF2-cIAP1/2 complexes. In the presence of an active immune check point, suppressing CD8^+^ T cells, this is of minor relevance **(A)**. Treatment with an immune checkpoint inhibitor restore CD8^+^ T cell activity empowering the later to produce TNF and to kill vulnerable tumor cells with low expression of FLIP proteins and low amounts of TRAF2-cIAP1/2 complexes via TNFR1 **(B)**. Activated CD8^+^ T cells may also produce IFNγ which can upregulate TWEAK in NK cells. NK cell produced TWEAK (or not shown exogenously added Fn14 agonists or SMAC mimetics) can sensitize tumor cells by interfering with the protective activities of the TRAF2-cIAP1/2 complexes making FLIP low expressing cells with proficient TRAF2 and cIAP expression killable by NK/CD8^+^ T cell derived TNF **(C)**. FLIP expression can be fully sufficient to protect against TNFR1-induced apoptosis. Thus, it is tempting to speculate that the antitumoral response of ICB and inhibition of the TRAF2-cIAP1/2 axis could be further enhanced by adding a drug reducing FLIP expression **(D)**. C8/R, caspase-8 and/or RIPK1; F, FLIP proteins; R1, TNFR1; T2/I, TRAF2-cIAP1 or TRAF2-cIAP2 complex.

To evade the immune system, tumors, among other things, hijack proteins, such as PD-L1 and CTLA-4, which are otherwise used by the body to restrict immune responses. Indeed, the introduction of biologics blocking the activity of such immune checkpoints has profoundly improved cancer therapy. Perhaps not unexpected in view of the central role of TNF in immune regulation, there is growing evidence that immune checkpoint blockade (ICB) comes along with increased TNF production (Figure 3). Indeed, expression of PD-L1 on tumor cells has been implicated in the inhibition of signaling pathways promoting TNF production (Kearney et al., 2017). The ICB-related upregulation of TNF has been associated with complex effects on ICB therapies including without claim on completeness:

i) TNF-induced upregulation of PD-L1 on tumor infiltrating lymphocytes (TILs) and monocytes and macrophages (Lim et al., 2016; Bertrand et al., 2017; Hartley et al., 2017; Wen et al., 2022),
ii) induction of activation induced cell death in CD8^+^ TILs. Indeed, there is solid evidence from studies with the TNF-inhibitor Enbrel (=TNFR2-Fc) and studies using TNF- and TNFR1-deficient mice that TNF-dependent AICD limits the efficacy of anti-PD1 and anti CTLA-4 ICB (Bertrand et al., 2017). There is furthermore initial preliminary evidence from a first clinical trial that triple therapy with anti-PD1, anti-CTLA-4 and a TNF blocker is safe and might increase the rate of responders (Montfort et al., 2021),
iii) therapy limiting immune-related adverse events including the induction of colitis (Perez-Ruiz et al., 2019) and
iv) killing of tumor cells by CD8^+^ T cell and/or NK cell derived TNF.

Indeed, with respect to the latter, TRAF2-cIAP1/2 depletion and sensitization for cytotoxic TNFR1 signaling seem to be again of particular relevance. TNFR1, caspase-8, FADD, RIPK1, the LUBAC components, TRAF2 and cIAP1 have all been identified in genome-wide Crispr/Cas9 screens as important factors involved in the control of tumor development by CD8^+^ T cells and NK cells. While TNFR1, FADD and caspase-8 have been crucially implicated in the killing of tumor cells by CD8^+^ T cells and NK cells, the LUBAC components were found to antagonize the latter (Kearney et al., 2018; Zhang et al., 2022). Intriguingly, TRAF2 and cIAP1 were furthermore found to be major sgRNA targets enhancing tumor cell killing under ICB (Vredevoogd et al., 2019). Accordingly, it was furthermore reported that TRAF2-deficiency and treatment with TWEAK or IAP antagonists sensitize for tumor cell killing by CD8^+^ T cell/NK cell-derived TNF (Kearney et al., 2017; Vredevoogd et al., 2019). Moreover, a comprehensive meta-analysis of genomic/transcriptional and clinical data of >1,000 patients treated with checkpoint inhibitors revealed that chromosomal loss of 9q34, the chromosomal region where the TRAF2 gene is located, is positively associated with a successful clinical response (Litchfield et al., 2021). In sum, these findings suggest that depletion of TRAF2 by Fn14 agonists and/or inhibition of cIAPs or the LUBAC by pharmacological drugs, such as IAP antagonists (= SMAC mimetics) or HOIPIN-8 (Katsuya et al., 2019) could improve the clinical outcome of ICB by promotion of the cytotoxic crosstalk between TNFR1 and TRAF2-depleting receptors (Figure 3). Since FLIP proteins protect from TNF-induced apoptosis independent from the TRAF2/cIAP axis, drugs interfering with FLIP expression have possibly the potential to further boost the antitumoral effects of ICB and TRAF2/cIAP inhibition (Figure 3). Indeed, many clinical and preclinical drugs have been found to interfere with FLIP expression.

## 6 The cytotoxic TNFR1-TNFR2 crosstalk in immune cells

First evidence for a promoting role of TNFR2 in TNF-induced cell death *in vivo* become already evident in the initial characterization of TNFR2-deficient mice which revealed reduced mortality in response to intravenous injection of low dose recombinant TNF and also reduced tissue necrosis upon subcutaneous TNF injection (Erickson et al., 1994). Experiments with neutralizing anti-TNF antibodies gave furthermore evidence for a role of endogenous TNF in activation induced cell death (AICD) in a T cell receptor transgenic mice model (Sytwu et al., 1996). Early on, it has also be found that TNFR2 engagement triggers cell death in splenic T cells activated *in vitro* with concavalin A and IL2 (Lin et al., 1997). In line with this, studies with purified anti-CD3/IL2-treated TNFR2-deficient CD8^+^ T cells revealed an involvement of TNFR2 in activation induced cell death (AICD) (Kim et al., 2009). A crucial role of TNFR2 in AICD was also evident for ovalbumin stimulated CD8^+^ T cells derived of TNFR2-defcient mice transgenic for the OT-I T cell receptor which recognizes a K^b^-presented ovalbumin peptide (Kim et al., 2009). Reduced AICD has also been observed for TNFR2-deficient CD4^+^ T cells and seem to result in enhanced CD4^+^ T cell transfer-induced colitis (Dayer Schneider et al., 2009). An involvement of TNFR1 in the AICD of CD8^+^ T cell has been furthermore observed in a model where infection with a mouse-adapted influenca virus triggers T cell driven lung injury. Infection-associated tissue injury in this model was enhanced in TNFR1-deficient mice and this correlated with a reduced capacity of the CD8^+^ T cells to undergo AICD (DeBerge et al., 2014). First side by side evidence that both TNFRs, and thus possibly the cytotoxic TNFR1-TNFR2 crosstalk, contribute to AICD came from studies with T cells of transmembrane TNF knockin mice. CD4^+^ T cells derived of these mice show enhanced cell death when supplemented with soluble TNF and this sensitizing effect disappears in a TNFR1-as well as in a TNFR2-defcient background (Müller et al., 2009). In line with a crucial role of the cytotoxic TNFR1-TNFR2 crosstalk in the AICD of CD8^+^ T cells, TRAF2 expression is higher in TNFR2-deficient CD8^+^ T cells under AICD conditions and retroviral expression of TRAF2 results in reduced AICD (Twu et al., 2011).

The relevance of the TNF-TNF receptor system for AICD of CD8^+^ T cells has been mainly investigated in murine models but studies with TNF blockers and primary T cells also argue for a role of TNF in the AICD of human CD8^+^ T cells (Otano et al., 2020). Similarly, CD4^+^ T cells derived of patients that severely suffered from COVID-19 showed strong AICD in response to treatment with spike 1-derived peptides which could be rescued by treatment with an anti-TNF antibody (Infliximab) or an anti-TNFR1antibody (Popescu et al., 2022).

It is important to keep in mind that TNF and its receptors are not only involved in the control of AICD but have also been implicated in T cell proliferation, T cell costimulation and T cell survival. Moreover, the available TNF concentrations in concert with the balance between soluble TNF (engages only TNFR1) and memTNF (engages both TNFR1 and TNFR2) along with the fact that T cells can produce TNF in an autocrine fashion could sculpt the net effect of TNF on T cells in a complex fashion. Last but not least, AICD of T cells does not only involve the TNF-TNFR system but is redundantly mediated by other DRs, especially CD95 but possibly also TRAIL (Alderson et al., 1995; Ettinger et al., 1995; Martinez-Lorenzo et al., 1998; Sedger et al., 2010). Therefore, the relevance of the cytotoxic TNFR-TNFR2 crosstalk in T cells is presumably highly context-dependent. Indeed, it has already been reported that the CD95L-CD95 system contributes to AICD of tumor infiltrating CD8^+^ T cells in anti-PD1/anti-CTLA-4 treated mice (Zhu et al., 2017), opening the possibility that TNF and CD95L act in this scenario in a redundant fashion.

In view of the fact that cytotoxic TNFR1 signaling is more redundantly controlled than death signaling by the other DRs (see above), it is tempting to speculate that the cytotoxic TNFR1-TNFR2 crosstalk becomes particular relevant in immune cells under pathophysiologic conditions when one or more of the inhibitory checkpoints of cytotoxic TNFR1 signaling are defective, e.g., due to mutations, or inhibition by virus- or pathogen-encoded factors inhibiting NFκB signaling or other cell death checkpoints with particular relevance for TNFR1. Indeed, in the Do-11.10 T cell hybridoma model of anti-CD3 antibody-induced AICD, inhibition of classical NFκB signaling resulted in a switch from CD95-dependent cell death to TNF-dependent cell death which could be rescued by ectopic A20 expression (Malewicz et al., 2003). Worth mentioning, TNF-dependent killing of anti-CD3 antibody-stimulated NFκB-inhibited Do-11.10 cells involves both TNFRs but has also a CD95-dependent component due to TNF-mediated CD95 induction (Malewicz et al., 2003).

## 7 Conclusion and perspective

Both TNFR2 and Fn14 recruit TRAF2 along with the TRAF2-interacting E3 ligases cIAP1 and cIAP2 and use these molecules for the stimulation of proinflammatory signaling pathways, e.g., the classical NFκB pathway. TNFR2-and Fn14-mediated recruitment of TRAF2 and the cIAPs is intimately linked to an impoverishment of the cytosolic pool of these molecules limiting their availability amongst others for TNFR1 signaling and regulated NIK degradation. In line with this, the most striking consequences of cytosolic impoverishment of TRAF2, cIAP1 and cIAP2 by TNFR2 and Fn14 are sensitization for TNFR1-induced cell death processes and activation of the alternative NFκB signaling pathway. While the physiological and pathophysiological relevance of “direct” TNFR2 and Fn14 signaling activities, such as classical and alternative NFκB signaling, have been intensively studied over the last two decades in animal models, the cytotoxic TNFR2-TNFR1 and Fn14-TNFR1 crosstalk have been poorly addressed *in vivo*. The exploding recognition of the broad relevance of TNFR1-induced cell death processes in infections and cancer development might now revitalize the interest in these in principle since over 25 years known crosstalk mechanism(s). Indeed, the expression patterns of TNFR1 (ubiquitous), TNFR2 (immune cells) and Fn14 (non-immune cells in injured tissue) and their ligands TNF and TWEAK, both expressed by activated myeloid cells, suggest that costimulation of TNFR1 and TNFR2 in immune cells and TNFR1 and Fn14 in non-immune cells are rather the rule then the exception when tissue homeostasis is disturbed. Future studies should now show to which extent TNFR2- and Fn14-mediated enhancement of TNFR1-induced cell contribute to pathophysiological scenarios in which TNFR1-induced cell death plays a crucial role. Since biologicals inhibiting or stimulating TNFR2 and Fn14 are already under preclinical investigation, it is tempting to speculate that new *in vivo* knowledge about the cytotoxic TNFR2/Fn14-TNFR1 crosstalk can straightforwardly foster new therapeutic strategies in the treatment of cancer and infections.

## Figures and Tables

**Table:** 

**AICD**	activation-induced cell death
**ADAM17**	a disintegrin and metalloprotease 17
**ASK1**	apoptosis signalling kinase 1
**CIA**	collagen-induced arthritis
**cIAP1/2**	cellular inhibitor of apoptosis 1/2
**CTLA-4**	cytotoxic T-lymphocyte-associated protein-4
**DAMP**	damage associated molecular pattern
**DD**	death domain
**tDED**	tandem death effector domain
**DISC**	death-inducing signalling complex
**DR3/4/5**	death receptor 3/4/5
**EAE**	experimental autoimmune encephalomyelitis
**FADD**	Fas-associated death domain protein
**Fn14**	fibroblast growth factor (FGF)-inducible 14
**FLIP-L/S**	long/short isoform of Fas-associated death domain-like interleukin-1β-converting enzyme (FLICE) like inhibitory protein
**GvHD**	graft-versus-host disease
**ICB**	immune checkpoint blockade
**IEC**	intestinal epithelial cell
**IKK**	inhibitor of kappaB kinase
**ILC2**	group 2 innate lymphoid cell
**LUBAC**	linear ubiquitin chain assembly complex
**LTα/β**	lymphotoxin-alpha/beta
**MAPK**	mitogen-activated protein kinase
**NFκB**	nuclear factor kappa-light-chain-enhancer of activated B cells
**NK cell**	natural killer cell
**PAMP**	pathogen-associated molecular pattern
**PD-L1**	programmed cell death ligand 1
**R/I**	reperfusion/ischemic
**RIPK1/3**	receptor-interacting protein kinase 1/3
**Treg**	regulatory T-lymphocyte
**TRADD**	TNFR1 associated death domain protein
**TRAF2**	TNF receptor-associated factor 2
**TRAIL**	TNF-related apoptosis inducing ligand
**TAK1**	transforming growth factor (TGF)-beta-activated kinase 1
**TNBS**	2,4,6-trinitrobenzene sulfonic acid
**sTNF**	soluble tumor necrosis factor
**memTNF**	membrane-bound tumor necrosis factor
**TNFR1**	TNF receptor 1
**TNFR2**	TNF receptor 2
**sTWEAK**	soluble TNF-like weak inducer of apoptosis
**memTWEAK**	membrane-bound TNF-like weak inducer of apoptosis
**UUO**	unilateral ureteral obstruction
**XIAP**	X-linked inhibitor of apoptosis protein
